# Preventive Metformin Monotherapy Medication Prescription, Redemption and Socioeconomic Status in Hungary in 2018–2019: A Cross-Sectional Study

**DOI:** 10.3390/ijerph18052206

**Published:** 2021-02-24

**Authors:** Csilla Nagy, Attila Juhász, Péter Pikó, Judit Diószegi, György Paragh, Zoltán Szabó, Orsolya Varga, Róza Ádány

**Affiliations:** 1 Public Health Administration Service of Government Office of Capital City Budapest, 1138 Budapest, Hungary; nagy.csilla@kmr.antsz.hu (C.N.); juhasz.attila@kmr.antsz.hu (A.J.); 2 MTA-DE-Public Health Research Group, University of Debrecen, 4028 Debrecen, Hungary; piko.peter@med.unideb.hu (P.P.); dioszegi.judit@med.unideb.hu (J.D.); 3 Institute of Internal Medicine, Faculty of Medicine, University of Debrecen, 4032 Debrecen, Hungary; paragh.gyorgy@med.unideb.hu; 4 Department of Emergency Medicine, Faculty of Medicine, University of Debrecen, 4032 Debrecen, Hungary; szabo.zoltan@med.unideb.hu; 5 Department of Public Health and Epidemiology, Faculty of Medicine, University of Debrecen, 4028 Debrecen, Hungary; varga.orsolya@med.unideb.hu

**Keywords:** socioeconomic status, metformin, redemption rate, preventive medication

## Abstract

This study was designed to characterize the spatial distribution of metformin medication used as first-line monotherapy for prevention of T2DM in relationship with the socioeconomic status (level of deprivation) and T2DM mortality at district level in a nationwide cross-sectional ecological study for the first time in a European country, Hungary. Risk analysis was used to estimate the relationships between socioeconomic status, characterized by tertiles of deprivation index, and mortality caused by diabetes, and metformin medication (both prescription and redemption) for the years of 2018 and 2019 at the district level. The spatial distribution of districts with a higher relative frequency of metformin prescriptions and redemptions showed a positive correlation with socio-economic deprivation. Significant association between the relatively high T2DM mortality and the highest level of deprivation could also be detected, but less-deprived regions with high T2DM mortality and low metformin utilization could also be identified. Although the statistical associations detected in this ecological study do not indicate a causal relationship, it is reasonable to suppose that the underuse of metformin medication may contribute to the unfavourable T2DM mortality in certain regions. Our findings underline the need for more effective preventive services including metformin medication to decrease T2DM morbidity and mortality burden.

## 1. Introduction

As the International Diabetes Federation (IDF) reports the number of people having type 2 diabetes mellitus (T2DM) more than tripled over the past 20 years; presently about 463 million adults are diagnosed with T2DM worldwide and 11.4% of all cause deaths are due to diabetes [1]. In addition to T2DM diagnosed in health care services the IDF estimates that in 2019 about 373.9 million individuals (7.5% in the adult population) was affected by prediabetes, a pathological condition characterized by impaired fasting glucose and/or impaired glucose tolerance, which indicates severely increased risk or early phase of the development of diabetes [2,3]. The rising incidence of T2DM across the world [1] increases the importance of evidence for effective ways to prevent it. Systematic review and meta-analysis [4] on prevention of T2DM in obese at-risk subjects, as well as the Cochrane Review on diet, physical activity or both for prevention or delay of T2DM and its complications in people at increased risk of developing T2DM [5] convincingly conclude that lifestyle change programs including body weight control and regular moderate exercise can lower the risk for developing T2DM. Nevertheless, it is generally accepted that if patients have not been able to control their prediabetes with lifestyle modifications, these interventions should be complemented with preventive medication [6]. Although prediabetes treatment is a subject of intense research and discussion, and many drugs have been found to be effective (as alpha-glucosidase inhibitors, orlistat, valsartan, thiazolidinediones, etc.), metformin which increases insulin sensitivity in the liver and skeletal muscles, delays or inhibits glucose absorption from the gastrointestinal tract is unanimously recommended as the first-line oral treatment [2,3,7,8]. Recommendations of the American Diabetes Association conclude that metformin has the strongest evidence base of pharmacological agents for diabetes prevention [9,10], in harmony with previous [11] and most recent findings [12].

In 2002, the DPP (Diabetes Prevention Program) showed that lifestyle intervention and metformin reduced the incidence of diabetes by 58% and 31%, respectively, compared with placebo over 2.8 years [13]. Although the DPP-based guidelines are almost generally accepted [14], in a retrospective cohort study Moin et al. demonstrated on a large national sample of insured, working-age adults that only 3.7% of patients with prediabetes were prescribed metformin over a three-year period (2010–2013). The prevalence of metformin prescription was higher (7.8%) among patients with a history of gestational diabetes or a BMI greater than 35 kg/m^2^, their conclusion was that fewer than 1 in 12 of patients, specifically identified by national guidelines, received metformin [15].

In a meta-narrative review Barry et al. pointed out that diabetes prevention programs neglect to consider the sociocultural environment in which affected people live [16] and by this way probably deepen inequalities in access to public health services. Regarding lifestyle modifying interventions against T2DM development Gray et al. demonstrated that patients from socioeconomically deprived groups were less likely to take up offers of these preventive measures [17]. In a qualitative study based on semi-structured interviews with 23 prediabetic patients from the most deprived practices of Sheffield (UK) it was showed that personal, social, and environmental factors hindered people to make lifestyle changes [18]. It is reasonable to suppose that socioeconomic factors may also have a reasonable effect on preventive medication with metformin, but no studies addressing directly this issue can be found.

In our previous studies we have demonstrated that while early death caused by cardiovascular diseases is in a strong positive correlation with socio-economic deprivation, preventive medication shows the opposite relationship for statin therapy against hypercholesterolaemia [19], for treatment with the new generation of antihypertensive medicines [20], as well as for antithrombotic preventive medication [21].

The aim of our present investigation was to characterize the spatial distribution of metformin medication used as first-line monotherapy for prevention of T2DM in relationship with the socioeconomic status (level of deprivation) and T2DM mortality at district level in a nationwide cross-sectional study for the first time in a European country, Hungary. In addition to the description of the effect of socioeconomic status on preventive metformin medication results are expected to help in the identification of gaps in preventive interventions which may contribute to the increased T2DM mortality in certain underserved regions.

## 2. Materials and Methods

In the present analysis the databases and methods used in our previous studies on preventive medication with statins, antihypertensive and antithrombotic drugs [19,20,21,22] were applied.

### 2.1. Data Sources

Hungary is divided administratively into 19 counties and the capital city Budapest. The counties are subdivided into 174 districts, while the capital Budapest into 23 districts.

The diabetes mellitus (ICD-10: E10–E14) mortality data at district level, for the years of 2018 and 2019 were obtained from the Hungarian Central Statistical Office, whilst the Central Office for Administrative and Electronic Public Services provided us population records for districts. Both mortality and population data were stratified by five-year age bands and sex.

The data of prescriptions for metformin and the data of redeemed metformin prescriptions were obtained from the National Health Insurance Fund Administration of Hungary for each primary healthcare practice for the entire years of 2018 and 2019. Metformin prescriptions and redemptions were collected only for patients who received metformin as monotherapy for prediabetes. The data were aggregated at district level and stratified similarly to the mortality data (i.e., by five-year age bands and sex).

### 2.2. Deprivation Index (DI)

Area based, district level Deprivation Index (DI) values are available to provide information about socio-economic status for 2011, the year of the last census in Hungary. The method for calculating DI was described previously [22] and successfully used in several studies [19,20,21,22,23,24,25,26]. Briefly, calculation of DI is based on seven municipality level socio-economic indicators (income, education level, unemployment rate, representation of one-parent families, that of large families, density of housing and car ownership) and was evaluated using the principal components analysis. The district and county level deprivation were determined using the population weighted average of DI.

### 2.3. Methods Used to Analyse Mortality and Medication Data

Since the free choice of family physician is a standard in Hungary, and the detailed population data and DI not available at practice level, reducing the misclassification risk all data were aggregated into district and county level. The districts were arranged into tertiles by DI, from the least deprived (tertile I) to the most deprived (tertile III).

Mortality data due to T2DM (ICD-10: E10-E14) for the 20+ age group for 2018 and 2019 combined were mapped at the district level, using hierarchical Bayesian-smoothed indirectly standardized mortality ratios [27]. The frequency of metformin prescriptions and redemptions in the 20+ age group for 2018–2019 was determined in relation to the national average, and their ratios for compliance were also depicted using the rapid inquiry facility—RIF [28]. The relationship between mortality and metformin medication with deprivation was defined by DI tertiles by applying the RIF. Chi-square tests for homogeneity and trend analysis were also implemented to investigate associations.

## 3. Results

### 3.1. The Spatial Distribution of Mortality Due to T2DM in Relation with Deprivation

**Figure 1:**
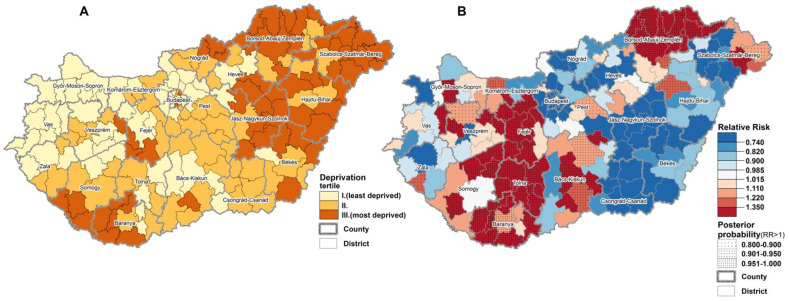
The territorial inequalities of deprivation (**A**) and mortality due to diabetes mellitus among people aged 20 years and over (**B**), at district level in Hungary, 2018–2019.

The geographical distribution of DI and mortality due to T2DM at district level in Hungary is shown in Figure 1. The districts of highest mortality risk were found at the southwestern (Somogy, Tolna, Baranya) and northeastern (Borsod-Abaúj-Zemplén) counties, as well as in an additional county (Fejér) located in the middle part of Hungary.

The majority of districts with the highest mortality risks seem to be localized in the more deprived areas of the country, and this is supported by the results of the risk analysis showing a significant association between the relative mortality and deprivation. The results of risk analysis showed a significant (no linear) association between the relative mortality due to T2DM and deprivation (χ^2^ homogeneity = 98.83, *p* = 0, χ^2^ linearity = 23.56, *p* = 0). In comparison with the country average T2DM mortality risk was found lower by approximately 6% in deprivation tertile I. (Relative Mortality_DI I._: 0.939; CI: 0.902–0.978) and by 7.5% in deprivation tertile II (Relative Mortality_DI II._: 0.925; confidence interval [CI]: 0.886–0.965). The mortality risk between tertiles I and II does not differ significantly, while in the most deprived tertile (III) the risk of mortality was significantly, by approximately 24% (Relative Mortality_DI III._: 1.236 [1.178–1.296], higher than the country average (Table 1).

Although the relationship between T2DM mortality and socioeconomic deprivation is not linear, T2DM mortality is significantly higher than the country average in the most deprived areas.

### 3.2. Prescription, Redemption and Redemption Rates of Metformin in Relation with Deprivation

In Hungary, 1,720,137.5 metformin prescriptions were prescribed per year in 2018–2019, and only 1,118,983.5, i.e., 65.1% of them (confidence interval (CI): 64.967–65.137) was redeemed. The frequency of prescription was 20.901/100 persons (CI: 20.879–20.923) and the frequency of redemption was 13.597/100 persons (CI: 13.579–13.615). These values served as reference in district/county level analysis.

**Figure 2:**
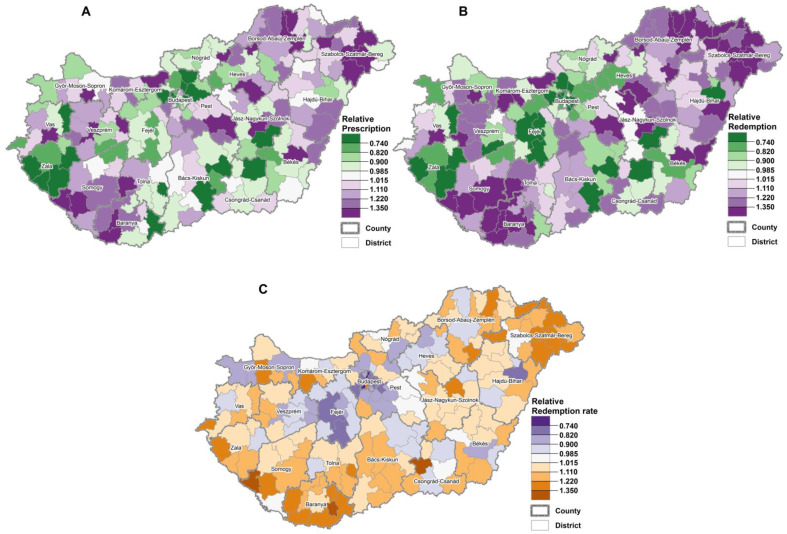
The territorial inequalities of relative frequency of prescription (**A**) and redeeming (**B**) of metformin and relative redemption rate (relative compliance) (**C**) in Hungary, 2018–2019.

The spatial distribution of districts with a higher relative frequency of metformin prescriptions and redemptions showed similar pattern as territorial inequalities. The frequency of prescription and redemption was higher in the northeastern, eastern and southwestern parts of Hungary (Figure 2A,B). In these areas, a higher relative compliance was found also (Figure 2C), while in the middle part of Hungary (Budapest (capital city), Pest and Fejér counties) lower relative redemption rates were observed (Figure 2C). It is noted that the districts with a lower relative redemption rates were showed like the territorial inequalities of areas with a lower frequency of metformin prescription (Figure 2C).

The risk analysis showed a significant positive linear association between relative frequency of prescriptions, that of redemptions, as well as redemption rate of metformin and deprivation (for prescription: χ^2^ homogeneity = 40,610.63, *p* = 0, χ^2^ linearity = 40,434.71, *p* = 0; for redemption: χ^2^ homogeneity = 63,371.83, *p* = 0, χ^2^ linearity = 62,787.78, *p* = 0; for redemption rate: χ^2^ homogeneity = 8513.32, *p* = 0, χ^2^ linearity = 7773.41, *p* = 0) (Table 2 and Table 3). In the areas with high deprivation (tertile III) 24.211 per 100 persons metformin prescriptions were found, i.e., the relative prescription frequency was higher than the national average by 16%. In the same tertile the frequency of redemptions was 17.297/100 persons, i.e., higher than national level by 27%, while the redemption rate was 71.5%, i.e., higher than national redemption rate by 10% (Table 2 and Table 3).

In the less deprived areas the frequency of metformin prescription was lower than the national average by 12% (18.376/100 persons), and that of the redemption by 17% (11.303/100 persons), while the redemption rate by 5.5% (61.462/100 persons) (Table 2 and Table 3).

In the most deprived areas the relative frequency of prescription, as well as that of redemption for metformin was significantly higher than the country average values, which indicate that higher T2DM mortality is not a consequence of less favourable preventive metformin medication and/or increased primary non-compliance, but other deprivation-related factors exist behind it.

### 3.3. Identification of Discrepancies in Metformin Preventive Medication Patterns among Counties with Different Socio-Economic Status

To demonstrate the variation in the relationship between socioeconomic status and T2DM mortality, as well as metformin medication, further analysis was carried out at county level.

Some counties were found where the relative mortality risk and frequency of metformin prescription and redeeming, as well as redemption rate were found to be higher than the national average, and these counties are localized in the most deprived region of the country (e.g., Borsod-Abaúj-Zemplén and Baranya counties). Contrarily, despite its unfavourable socioeconomic status a single county with lower relative mortality risk and lower metformin medication than the national average was also detected (Hajdú-Bihar county). Furthermore, an area was identified among counties in the less deprived tertile with significantly higher T2DM mortality, but with significantly lower relative metformin medication (Fejér county and Eastern part of Tolna county—forming together a cluster in the midwestern part of Hungary). In general, it can be accepted that socio-economic deprivation is associated with increased T2DM mortality and higher level of metformin utilization, but exceptions to this statistical correlation in both respects can be identified (Table 4).

The above findings indicate that increased mortality caused by T2DM independently of the socioeconomic status of the regions affected may relate to insufficient preventive metformin medication.

## 4. Discussion

The latest analysis carried out by using the Global Burden of Disease (GBD) database for 2017 [29] showed that age-standardized mortality caused by T2DM is significantly higher (by 70%) in the Central European (CE) countries (11.0/100,000 population) than in the Western European countries (7.7/100,000). The age-standardized death rate for T2DM in Hungary is 25.81/100,000, i.e., more than double of the average of CE countries. Findings in our present study clearly indicate that in general T2DM mortality is about 30% higher than the country average in the most deprived regions of the country, but significantly higher mortality could be also observed in districts/counties among the least deprived regions too. Our results in general are in good harmony with observations in the U.S. from a nationally representative cohort study that low socio-economic status indicated either by family income below poverty level or by low education level was associated with a two-fold or higher mortality from T2DM [30], but findings representing exemptions, i.e., clusters with high mortality in the less deprived regions, as well as low mortality rates in highly deprived ones, suggest that other than socioeconomic status-related factors may also contribute to the increased T2DM death rates.

In the present study a positive association between socioeconomic status and metformin medication could be detected. Former study on US prediabetes patients found that metformin use did not vary by race, poverty-to-income ratio, or education [31], but the prevalence of metformin medication among those with prediabetes was as low as 0.7%.

Among our results it should be highlighted that the spatial distribution of metformin prescription and redemption only partially overlapped the districts/counties with high T2DM mortality. Our findings suggest that although high T2DM mortality in the most deprived regions cannot be linked neither with underuse of metformin medication nor less favourable primary compliance (i.e., redemption of prescriptions), but districts can be identified where the low metformin prescription/redemption rates are associated with high T2DM mortality rates. Studies from the US suggest that only about half of the family physicians have positive attitude towards prediabetes as a diagnostic construct [32] and even if the academic family practitioners (clinicians) consider prediabetes screening, diagnosis and management are important health issues, according to the electronic charts of the patients clinicians suggested most patients with prediabetes counselling on physical activity, but less than one third of them reported prescribing metformin [33]. Similar study was not carried out among Hungarian general practitioners (GPs), but on the basis of the results of our survey by the involvement of 34 Hungarian GPs it was shown that in case of 64% of individuals registered in the GPs’ practices the regular health check legally specified as once every three years (51/1997. Decree of the Ministry of Welfare) was not completed. The fasting blood glucose measurement whose once every two years frequency is also legally specified is performed only in 34% of practice clients. It is reasonable to suppose that the missing financial incentives for prevention services in primary care strongly hampers service provision regarding prediabetes screening as well [34]. Although detailed guideline for practitioners on diabetes screening was published by the Hungarian Diabetes Federation almost ten years ago [35], not simply the prediabetes screening is insufficient, but even monitoring the achievement of fasting blood glucose and HbA1c target values in case of diabetic patients is largely incomplete (proportion of uncontrolled subjects was 43–45% in 2016) [36]. To improve prediabetes primary prevention, screening and management the concluding remark of the latest Cochrane review analysed the results obtained in fifteen studies on the effect of preventive medication with metformin in comparison with that of lifestyle modifying interventions against T2DM development: “When compared to standard diet and exercise metformin slightly reduces or delays development of diabetes.” [37].

The method we used in our present study to analyse the relationship between deprivation and preventive medication in comparison with the mortality caused by the disease targeted can be applied not only to investigate the effect of socio-economic status on different diseases and service provisions, but also to identify severe gaps between mortality and services necessary to decrease morbidity and mortality related to diseases examined. Regarding the relationship between socioeconomic status and metformin utilization for T2DM prevention, as well as between T2DM mortality and preventive medication by metformin no data are available in the literature. To the best of our knowledge, our present study is the first comprehensive report on the association of socio-economic deprivation and metformin medication in comparison with T2DM mortality figures on the entire population of a country with very high T2DM mortality among European countries. In addition to the description of the significant positive association between deprivation and metformin utilization in general, gaps in preventive medication which may be linked to T2DM mortality much over the country average are also identified. However, some limitations need to be considered in the interpretation of our findings. In our observational study with the methodology of cross-sectional and ecological studies, the statistical associations detected do not indicate a causal relationship, and the relationships observed between deprivation and mortality, as well as deprivation and preventive medication at the population level, may not be linked with mortality and preventive medication in individuals [38]. It is worth mentioning among the limitations that valid T2DM morbidity data at district level are not available in Hungary, so the spatial distribution of morbidity burden caused by T2DM could not be considered. The high prevalence of health behaviour risk factors (such as unhealthy nutrition, high consumption of alcohol, physical inactivity) contributing to the development of T2DM is a severe problem in Hungary [39,40] and these factors were unmeasured confounders in our study. In addition, the deprivation index calculation is based on the last census (delivered in 2011) data, while T2DM mortality and metformin utilization data are from the years of 2018 and 2019. Obviously as we move away from the year of the census the precision of DI values might decrease, so this gap in time may result in some uncertainty in the interpretation of DI values and their effects on any variables. As we described in connection with the development and introduction of DI [22] the indicators at the municipality/district level may become less appropriate over time as populations move and areas change in character; a new census may provide information to update the required indicators.

## 5. Conclusions

Deprivation was found to be a significant determinant of mortality due to T2DM in Hungary that is in harmony with the literature. Although T2DM mortality is significantly higher in the most deprived regions it cannot be simply linked neither with underuse of metformin medication nor less favorable primary compliance. The method applied in this study to assess the relationship between deprivation and preventive medication in comparison with the mortality caused by a particular disease highlights regions/populations with sufficient access to adequate treatment and white spots between mortality and services necessary to decrease morbidity and mortality related to that disease. Although the statistical associations detected in this ecological study do not indicate a causal relationship, it is reasonable to suppose that assessing the efficacy of preventive services including preventive medication is the key to unlock better health outcomes and healthcare performance.

## Figures and Tables

**Table 1:** The risk of mortality due to diabetes mellitus for 20–X age group at district level by deprivation tertiles, compared to the national average mortality in Hungary, 2018–2019.

DI Tertiles	Average Observed Cases/Year	Relative Mortality [95% CI]
I. (least deprived)	1157.5	0.939 [0.902–0.978]
II.	1070.5	0.925 [0.886–0.965]
III. (most deprived)	850.5	1.236 [1.178–1.296]

**Table 2:** Relative frequency of prescription, redemption, and the relative redemption rate of metformin per 100 persons aged 20+ years at district level by deprivation tertiles, compared to the national average, in Hungary, 2018–2019.

	DI Tertiles		
	I. (Least Deprived)	II.	III. (Most Deprived)
Average number of prescription/year	592,326.5	667,952	459,859
Relative frequency of prescription [95% CI]	0.88 [0.878–0.881]	1.027 [1.025–1.029]	1.16 [1.157–1.162]
Average number of redemption/year	364,427	426,139	328,417.5
Relative frequency of redemption [95% CI]	0.832 [0.83–0.834]	1.007 [1.005–1.009]	1.274 [1.27–1.277]
Relative redemption rate [95% CI]	0.945 [0.943–0.947]	0.981 [0.978–0.983]	1.099 [1.096–1.102]

**Table 3:** Frequency of prescription, redemption, and relative redemption rate of metformin by deprivation tertiles at district level, in Hungary, 2018–2019.

	DI Tertiles		
	I. (Least Deprived)	II.	III. (Most Deprived)
Frequency of prescription (per 100 persons aged 20+ years) [95% CI]	18.376 [18.350–18.403]	21.466 [21.439–21.494]	24.211 [24.175–24.248]
Frequency of redemption (per 100 persons aged 20+ years) [95% CI]	11.303 [11.281–11.326]	13.696 [13.671–13.721]	17.297 [17.263–17.331]
Redemption rate (%)	61.462 [61.374–61.550]	63.784 [63.702–63.865]	71.485 [71.393–71.578]

**Table 4:** Relative mortality due to diabetes mellitus and relative frequency of the prescription and redemption, as well as relative redemption rate of metformin of the population aged 20+ years, in selected counties of Hungary, 2018–2019.

County	Relative Mortality	Relative Frequency of Prescription	Relative Frequency of Redemption	Relative Redemption Rate	Deprivation Index
					(Tertile)
Borsod-Abaúj-Zemplén	1.887	1.177	1.258	1.068	0.834537 (III.)
	[1.753–2.029]	[1.173–1.182]	[1.252–1.264]	[1.064–1.073]	
Baranya	1.532	1.173	1.378	1.174	0.05541 (III.)
	[1.382–1.694]	[1.167–1.179]	[1.37–1.385]	[1.167–1.181]	
Hajdú-Bihar	0.883	0.99	0.975	0.986	0.45887 (III.)
	[0.782–0.993]	[0.985–0.994]	[0.969–0.98]	[0.98–0.991]	
Fejér	2.524	0.916	0.766	0.837	−0.45942 (II.)
	[2.332–2.728]	[0.911–0.921]	[0.761–0.772]	[0.831–0.843]	
Eastern-Tolna	2.116	0.936	1.087	1.163	−0.73643 (I.)
	[1.842–2.42]	[0.928–0.944]	[1.076–1.097]	[1.151–1.174]	

## Data Availability

Publicly available datasets were analyzed in this study. This data can be requested from: Hungarian Central Statistical Office (http://www.ksh.hu/?lang=en (accessed on 20 February 2021)); Tasks of the National Health Insurance Fund of Hungary (http://www.neak.gov.hu/ (accessed on 20 February 2021)).
